# The experience of family caregivers of ventilator-assisted individuals who participated in a pilot web-based peer support program: A qualitative study

**DOI:** 10.1177/20552076221134964

**Published:** 2022-11-25

**Authors:** Marina B. Wasilewski, Kristina M. Kokorelias, Mika Nonoyama, Craig Dale, Douglas A McKim, Jeremy Road, David Leasa, Anu Tandon, Roger Goldstein, Louise Rose

**Affiliations:** 1 St. John's Rehab Research Program, 282299 Sunnybrook Research Institute, Toronto, Ontario, Canada; 2 Rehabilitation Sciences Institute, Temerty Faculty of Medicine, 7938 University of Toronto, Toronto, Ontario, Canada; 3 Faculty of Health Sciences, 85458 Ontario Tech University, Oshawa, Ontario, Canada; 4 Lawrence S. Bloomberg Faculty of Nursing, 7938 University of Toronto, Toronto, Ontario, Canada; 5 Clinical Epidemiology Program, 10055 Ottawa Hospital Research Institute, Ottawa, Ontario, Canada; 6 Division of Respiratory Medicine, 8166 University of British Columbia, Vancouver, British Columbia, Canada; 7 Schulich School of Medicine & Dentistry, 6221 Western University, London, Ontario, Canada; 8 Division of Respiratory, 71545 Sunnybrook Health Sciences Centre, Toronto, Ontario, Canada; 9 Respiratory Medicine, 27375 West Park Healthcare Centre, Toronto, Ontario, Canada

## Abstract

**Introduction:**

Family caregivers play an important role supporting the day-to-day needs of ventilator-assisted individuals (VAIs) living at home. Peer-to-peer communication can help support these caregivers and help them sustain caregiving in the community. Online peer-support has been suggested as a way to help meet caregivers’ support needs.

**Methods:**

A qualitative descriptive approach was used to elicit the perspectives of support received from caregivers who participated in a pilot web-based peer support program from October to December 2018. Data were collected through the transcripts of weekly online peer-to-peer group chats. Data were analyzed using an integration of thematic and framework analysis.

**Results:**

In total, eight caregivers and five peer mentors participated in the pilot. All five mentors and four of the caregivers participated in the weekly chats. We identified three themes, a) The experience of caregivers is characterized by unique challenges related to the complexity of VAI care including technology; b) Mentors and caregiver participants reciprocally share support; c) Despite hardships, there are things that make caregiving easier and joyful.

**Discussion:**

Our results add to the growing body of evidence pointing to the importance of online communities for supporting vulnerable caregivers. The reciprocal element of peer support, where trained mentors and untrained participants both benefit from support, can help sustain peer-support interventions. Despite the challenges of providing care to a VAI, there are facilitators that may help ease the caregiving experience and caregivers can benefit from ongoing support that is tailored to their needs along the caregiving trajectory.

## Introduction

Neuromuscular disorders, such as amyotrophic lateral sclerosis (ALS), muscular and myotonic dystrophy, and multiple sclerosis result in progressive respiratory failure that necessitates mechanical ventilation.^1,2^ Policymakers report increased interest in helping ventilator-assisted individuals (VAIs) return to, or remain in the community, rather than in institutional care settings due to cost savings as well as benefits in terms of health- related quality of life for VAIs.^2,3^ Family caregivers, often spouses, parents, or children of elderly parents play an important role supporting community integration and promoting quality of life of adults who experience mechanical ventilation.^4^ The physical care provided by caregivers matches and often exceeds the receipt of formal care (e.g., from personal support workers [PSWs]), with levels of formal support varying internationally.^2,5,6^ Family caregivers of VAIs constitute a largely unexplored population within health research. The limited research that exists indicates that caregivers have few supports and are often unprepared to provide care at home.^7–9^

Provision of care to VAIs can decrease caregivers’ own well-being, including their social, emotional, and physical health.^6,10,11^ Although mounting research has addressed the needs of caregivers of other patient population in terms of assistance with their role and importance of mitigating declining health,^12–14^ caregivers of VAIs have been unrepresented in this evidence. Furthermore, in many jurisdictions, standard clinical practice includes minimal training or preparation of VAI caregivers for the intensive support role they take on in the community.^9,15^ A meta-review of systematic reviews of interventions to support caregivers concluded that there is no one size fits all intervention that can support all caregivers. Caregivers vary in their needs and ability to benefit from different types of interventions.^16^ Thus, it is important to understand the experiences of specific caregiving populations to better tailor support to meet their needs.

Social support may play a protective role in helping caregivers sustain their well-being, and ultimately, their ability to provide care.^17^ Social support has consistently been associated with fewer negative and more positive caregiver outcomes.^18,19^ Caregiving peers may serve as a useful resource of providing social support.^20^ Caregiving peers are those with the lived experience of caring for the same population.^21^ With the increased uptake of technology, a growing number of caregivers use the Internet to gather illness-specific information and find caregiving peers.^22,23^ As a result, online peer-support programs for family caregivers of individuals living with disability have been implemented and evaluated for a variety of illness populations.^24–26^ However, no studies to date have explored online peer support for family caregivers of VAIs living at home.^27^

Therefore, we developed a multicomponent online peer support program for caregivers of VAIs living at home. This program involved interaction with trained peer mentors (i.e., previous or current family caregiver for a community-residing VAI) and caregiver participants. The program was delivered via a website that included informational links, private chat, and a discussion forum. Mentor training and program development details can be found in our published protocol.^27^ Although it is important to capture the experiences of caregivers using online peer-support programs, these experiences are currently underrepresented in the published evidence base. To help inform the development of future support interventions, our study aimed to generate an understanding of the experiences of caregivers of VAIs with the pilot version of our online peer support program as well as their perceptions of the support they received in the program (e.g., quality of support, influence on their caregiving experience).

## Methods

### Design

A qualitative descriptive approach was employed.^28^ A qualitative approach was chosen to enhance our understanding of the experiences of caregivers with the peer support program and their perspectives on the support received from peers. This study reports on data from the pilot of the web-based peer support program.^27^

The Standards for Reporting Qualitative Research were followed throughout the reporting of this study.^29^ Research ethics approval was obtained from the University of Toronto, Queen's University, Sunnybrook Health Sciences Centre, and West Park Healthcare Centre.

### Participants and recruitment

Purposeful and snowball sampling were used to recruit participants.^30^ Family caregivers (i.e., friends, family members or neighbours of VAIs) were recruited through the Ventilation Equipment Pool (Kingston, ON, Canada), the long-term and home ventilation clinics of West Park Healthcare Centre (Toronto, ON, Canada), clinician experts, professional societies, patient advocacy groups (e.g., Muscular Dystrophy Canada), and through Twitter. Participants were eligible for the study if they: (1) were at least 18 years of age; (2) self-identified as a family caregiver for a VAI living at home; (3) able to speak and read English; and (4) had access to a computer (with video and microphone) and a high-speed internet connection. Participants who volunteered to act as peer mentors had to meet the above criteria in addition to being available for the mentor training sessions (details of the training sessions can be found in our published protocol^27^). Participants were excluded if they were experiencing severe depression during the study period.

### Data collection

Data was collected from secure group text chats that took place as part of the online peer support program. Participants were made aware that the transcripts of the online text chats would be downloaded and analyzed as research data. The weekly online discussions occurred from October 18, 2018 to December 13, 2018 (9 weeks for approximately an hour each). Chat transcripts were downloaded from the website. The first author (MBW) or another member of the research team (LR, MN) moderated weekly chat sessions. Each weekly chat had a specific topic (e.g., self-care, navigating the healthcare system) and three to four “topic questions” to guide the discussion in addition to two “ask-the-expert” session with two healthcare providers (i.e., open forum with no pre-determined questions that allowed participants to ask their own questions of interest) (Table 1).

**Table 1.** Weekly chat Schedule

Topic	Probing Questions
Week 1 (Oct 18, 2018)	
Caring for your loved one	**Question 1:** What are your top priorities in caring for your loved one? **Question 2:** What (if any) challenges do you face in caring for your loved one? **Question 3:** What helps you in providing care to your loved one?
Week 2 (October 25, 2018)	
Setting boundaries	**Question 1:** What boundaries (personal, caregiving, etc) are important to you? Why? **Question 2:** What do you find easy/straight-forward about boundary setting? **Question 3:** What do you find challenging about boundary setting?
Week 3 (Nov 1, 2018)	
Navigating the system	**Question 1:** What are some challenges you’ve faced with the system? **Question 2:** What has helped you navigate the system? **Question 3:** What could improve your experience with the system and help you navigate it?
Week 4 (Nov 8, 2018)	
Caregiving and other relationships	**Question 1:** How has caregiving affected your other valued relationships? **Question 2:** How do you navigate/manage issues that arise in other relationships? **Question 3:** What are some positive changes or experiences you’ve had with other relationships?
Week 5 (Nov 15, 2018)	
Managing caregiver stress	**Question 1:** What are some stresses you face as a caregiver? **Question 2:** How do you manage stress and engage in ‘self-care’? **Question 3:** What are some challenges you face in managing stress and caring for yourself? **Question 4:** What makes caring for yourself easier?
Week 6 (Nov 22, 2018)	
Ask the Expert	*Open discussion with a Staff Respirologist*
Week 7 (Nov 29, 2018)	
Social engagement	**Question 1:** What types of social activities are important to you? **Question 2:** What challenges do you face in maintaining your social engagement? **Question 3:** What helps you to maintain your social engagement?
Week 8 (Dec 6, 2018)	
Finding joy in caregiving	**Question 1:** What are some successes or positive experiences you’ve had while providing care for your loved one? **Question 2:** What helped facilitate those positive experiences? **Question 3:** What were some challenges or hindrances to those positive experiences happening?

### Data analysis: theoretical framework

Peer support resources and programming can be optimized by identifying key functional components of effective support and applying them flexibly according to regional needs, specific populations, and varying health systems.^26^ To this end, Peers for Progress (a program developed by the American Academy of Family Physicians Foundation) developed a conceptual framework of how peer support programs might be implemented in varied settings.^26^ According to the Peers for Progress framework, the four key functions of support from peers are: (*a*) assistance in daily management; (*b*) social and emotional support to encourage management behaviors and coping with negative emotions; (*c*) linkage to clinical care and community resources; and (*d*) continual support^26^ (p.64). By understanding peer support according to these functions, evidence can be more easily mobilized to promote what is known about peer support in specific populations (e.g., VAI caregivers) in order to enhance its application and impact.^26^

### Data analysis: process

Data were analyzed using an integration of thematic^31^ and framework analysis.^32^ Thematic analysis was selected to help uncover overarching messages in the data and framework analysis was used to identify perceptions of support as it relates to the Peer for Progress framework.^26^ Two authors (MBW and KMK) first reviewed the transcripts several times to familiarize themselves with the data.^31^ Beginning with open coding, a subset of the transcripts were coded by MBW and KMK to generate an initial list of codes. These codes included the four key aspects of peer support programs identified in the Peers for Progress framework,^26^ as well as codes that described preliminary insights from the data.

MBW and KMK met to discuss their initial thoughts on the data and determine an exhaustive coding scheme. KMK applied this coding scheme to all the transcripts and then combined similar codes into larger categories. Next, codes and categories were analyzed and synthesized into larger categories based on the research objectives.^33^ MBW and KMK reviewed the raw, coded, and categorized data to develop preliminary themes. The preliminary themes were shared with the larger researcher team, who contributed to clarifying and refining the themes generated. KMK then gave each theme a title that reflected the data. Triangulating between multiple researchers enhanced the quality of our data analysis, thereby ensuring greater trustworthiness of the interpretations presented. f.^34^

## Results

The pilot study included at total of thirteen participants, with n = 5 peer mentors and n = 8 caregiver participants. On average, peer mentors were 54 years old; caregiver participants were 60 years old. All peer mentors were female, only one caregiver participant was male. On average, peer mentors and caregiver participants had provided 14.5 and 9.7 years of care, respectively. Detailed participant characteristics are reported in Table 2. All five of the peer mentors participated in the weekly chats but only four of the eight caregivers participated.

**Table 2.** Characteristics of participants, n.

Characteristic	Caregivers (n = 8)	Peer-Mentors (n = 5)
Gender		
Female	7	5
Relationship to VAI		
Spouse	5	3
Parent	2	2 0
Child	1	
Residence		
Urban	5	4
Rural	3	1
Marital status		
Married	7	2
Single	1	1
Widowed		1
Divorced		1
Highest level of education		
University	3	3
Post-graduate (no diploma/degree)	3	1
College	2	1
Employment status		
Self-Employed	1	2
Disability	1	1
Part-time	2	1
Full-time	2	1
Retired	2	0
Income (CAD)		
10-29K	1	2
30-39K	2	0
40-49K	1	1
50-59K	1	1
100-149K	1	0
150K +	1	1
Unspecified	1	0
Method of ventilation for VAI		
Tracheotomy	4 31	3
Face and nasal mask		2
Diaphragmatic pacer		0
24 Hour ventilation requirement for VAI		
Yes	2	4
Self-rated health		
Very good	1	3
Quite good	2	1
Neither good nor poor	4	0
Quite poor	0	1
Poor	0	0
Unspecified	1	0
Mean age of VAI in years (SD)	55.9 (σ:11.8)	42.3 (σ: 23.7)
Mean duration of care in months (SD)	116.5 (σ:108.2)	174 (σ: 116.7)

We identified three overarching themes that each contained several subthemes. Theme 1: “The experience of caregivers is characterized by unique challenges related to the complexity of VAI care including technology” (*Subthemes*: (a) Hypervigilance is required; (b) Boundary-setting is difficult; and (c) Role overload ensues). Theme 2: “Mentors and caregiver participants reciprocally share support” (*Subthemes*: (a) Assistance in applying disease management in daily life; (b) Emotional and social support; and (c) Need for ongoing support). Theme 3: “Despite hardships, there are things that make caregiving easier and joyful” (*Subthemes*: (a) Tangible and emotional support from others; (b) Compassionate care; and (c) Positive outlook on the caregiving journey). To ensure anonymity, we use participants’ unique identifiers (CG-caregiver; MN-mentor) for each quote.

### Theme 1: the experience of caregivers is characterized by unique challenges related to the complexity of VAI care including technology

Participants described a number of challenges they faced while caring for a VAI, including a lack of access to health and community resources, increased vigilance, struggles with boundary setting, and role strain (i.e., overwhelmed in their current role or at odds with their other relationship identities). In many instances, these challenges uniquely stemmed from the use of ventilator technology. The following sub-themes are described in detail (a) Hypervigilance is required; (b) Boundary-setting is difficult; and (c) Role overload ensues.

#### Subtheme A: hypervigilance is required

As a result of the lack of expertise in ventilator-related care amongst community professional healthcare providers, participants described how they had to remain vigilant at all times, even when the VAI is receiving care from others. Some caregivers explained that training and the hypervigilance required to supervise healthcare professionals took time away from their other meaningful activities such as employment or socializing with friends. One participant shared:

“*At most I can sit in the hall outside his room but I need to see him to make sure he doesn't have trouble breathing from a mucus plug. I would take the time to be with friends if I could”* (CG06) Another wrote: “*I think of this management of nurses and PSWs like parenting. You begin with learning/training. You spend time with them until they can do it themselves*” (MN06).

Participants described that hypervigilance negatively impacted their wellbeing. One participant shared: *“Health care professionals should know better and they can cause a lot of frustration. My biggest problem is staffing which translates into things like being physically exhausted”* (MN05).

Participants also described a lack of continuity of care between hospital and community settings. As a result, caregivers were often required to perform roles without formal training (e.g., assistance with medical care) that would be expected of paid healthcare professionals. This was especially challenging for caregivers in our study due to the complexity of ventilator technology and gravity of its life-preserving purpose. Participants described that community support was not readily available, especially in times of emergency. Similarly, participants described difficulties with receiving appropriate ventilators once out of the hospital. One caregiver shared: *“My husband needed the respirator to live. It's a bit scary when help is 2 h away”* (MN05). Participants also highlighted that a lack familiarity with ventilator-related care poses a challenge for community residing caregivers. One participant wrote:

“*I have had lots of pressure to change ventilators because they no longer want to supply/maintain the ventilator he is on now. This is a huge change since we have been on his ventilator for 11 years…and they think it is as easy as a phone call to me and it's all changed. So it's become a very uncomfortable situation and they are even less helpful now since we are refusing to change…this is the types of frustration that seems normal around lack of understanding of ventilation in the community even from professionals”* (MN08).

Despite the challenges participants shared, they did not explicitly identify needs for their own support that might counteract the need to be hypervigilant. Rather, they were more concerned about meeting the needs of the care recipient. For example, when asked about support needs caregivers would like to receive, one participant shared *“To see that all of his needs are met. Physical ones first of course, but also emotional and social need.”* (MN01)

#### Subtheme B: boundary-setting is difficult

Due to a perceived lack of appropriate support from health and community services, participants described difficulties with setting personal boundaries for their own time and participation in care. One participant wrote: “*The Director of the [local health network] told me I need to be available 24/7 but I told her I need to sleep, eat, shower, drive, etc. She said it didn't matter as my husband's power of attorney I had to be available all the time. How do I set boundaries up for that?”* (CG08). As a consequence of not being able to set boundaries, all participants described prioritizing the care of the VAI over themselves.

#### Subtheme C: role overload ensues

Most participants identified that not being able to detach from their caregiver role (often due to hypervigilance with ventilator technology) prevented them from having time to focus on their other relationships (e.g., friendships), fulfilling their caregiving role in conjunction with other societal roles (e.g., employee) as well as maintaining relational identities to the care recipient (i.e., husband/wife identity, parent/child identity). Participant quotes illustrating this included: *“Intimate relationship is non-existent, I have lost the wife part”* (CG02), *“I think caregiving is taking up a lot of my time and attention. I think I would have been a better Mom”* (CG06) and *“It's hard to imagine the change of role for a wife etc since for me I am always a mom so it doesn't seem like I had to figure that tricky part out…moms are caregivers”* (MN08).

### Theme 2: mentors and caregiver participants reciprocally share support

While a great deal of the support exchanged amongst peer support program participants was overt and explicit (e.g., informational support), much of the support was implicit (e.g., affirming things others said with a ‘yes’ or ‘I understand’). Participants discussed support as it pertained to three subthemes: a) Assistance in applying disease management in daily life; b) Emotional and social support; and c) Need for ongoing support.

#### Subtheme A: assistance in applying disease management in daily life

Both peer mentors and caregivers shared their experience of providing different aspects of care, such as health system navigation, assisting with basic activities of daily living (e.g., feeding) and instrumental activities of daily living (e.g., housekeeping). Through the sharing of past experiences, participants described learning how to apply different care techniques and strategies to their daily lives. Some participants did not feel the healthcare professionals they interacted with had the appropriate expertise to support them, and therefore appreciated hearing about the lived experiences of peers for suggestions on how to manage care. As one participant explained, *“Peer support is essential”* (MN05). Participants identified that it was only through their lived experience that they learned how to provide care and were happy to share this experience with others. *“All of it changes with time and unfortunately it gets more and more difficult…you only learn this as you go through it or hear from others who go through it”* (CG08). Participants also shared other ways they found support, such as through websites. In response to one question about medical care by one of the peer mentors, a caregiver participant wrote: *“I find medical websites helpful for getting factual medical information. I didn't know what pages to go to at first”* (CG06).

Participants shared their experiences with obtaining clinical care as a way of enabling others to learn from their experiences. For example, one peer mentor asked: *“If you don't mind sharing, I’m curious what vent people are using. I’m only asking because I’m interested to know what types are supported in the community since we will need to change….”* (MN08). Another example is when a caregiver was describing the difficulty of finding appropriate supports, a peer mentor responded with *“The biggest thing that has helped me, is really learning the system…I found an advocate within our [local health network] that I asked lots of questions to and asked for documentation and the more I found out the more I learned to make the system work for me….I learned a no is never really a no!”* (MN08).

#### Subtheme B: emotional and social support

In addition to the exchange of information, participants often asked one another to share similar experiences. Participants explained that as they did not have many opportunities for social support and frequently were the ones training healthcare professionals on ventilator-related care, they were unsure about some of the decisions they had made. Similarly, participants described that it was difficult to connect with some of their friends. One participant explained: *“I don't have a friend who understands”* (CG03). Participating in the peer support program offered participants a way to ask questions that required answers stemming exclusively from lived experience. One example is a participant who was inquiring about others’ experiences with seeking respite care: *“I am asking for respite care because after 15 years it is necessary to maintain a healthy life. I keep saying we need respite, and they continue to say ‘No’. Does anyone else have this situation?”* (CG06). Participants also offered affirmational support by constantly providing each other with praise and re-enforcement. For example, *“My heart goes out to you. That must have been terrifying. Good on you, you managed well”* (CG06) and *“I am really impressed”* (CG02).

#### Subtheme C: ongoing support

Participants did not explicitly describe any ongoing support experienced or provided by other peers. However, participants described that despite having experience with caregiving for many years, there were still areas of support that could be better met over a prolonged period of time. For example, participants described requiring new information, emotional support and assistance with system navigation as the caregiving trajectory continued. One participant shared *“I was doing okay, but the transition to adult care is very challenging as services are fewer and they are all spread out in time”* (CG08).

### Theme 3: despite hardships, there are things that make caregiving easier and joyful

Participants described several factors that had helped them in their caregiving role, despite the challenges they experienced. These facilitators of care were described by participants as making the caregiving journey easier and more joyful. The following subthemes capture the specific facilitators discussed by caregivers: a) Tangible and social support from others; b) Compassionate care; c) Positive outlook on the caregiving journey.

#### Subtheme A: tangible and emotional support from others

All of the participants described themselves as the primary source of support for the VAI. However, some participants shared that they did occasionally receive help from other family members or friends who they have trained to support the VAI. One participant explained: *“I have a small number of family and friends who I’ve trained to be able to care for [Spouse] so I can get away sometimes for a few hours if it is a time of day when I don't have a nurse here”* (MN02). Many participants also emphasized the importance of having access to tangible supports such as homecare workers (e.g., PSWs) or appropriate physicians to help support the VAI with medical care. For example, in response to a conversation around tangible support that would be helpful to caregivers, one replied *“Respite care in a chronic care unit with respiratory therapists and nurses that know how to care for a person on a vent.”* (CG01).

In addition to tangible support that allowed the participants to get respite, participants also described how family and friends provided emotional support to them and the care recipient. When describing the emotional support friends provide, one participant pronounced *“Support from anyone can bring instant relief!”* (MN08).

### Subtheme B: compassionate care

While discussions around the expertise of healthcare professionals in the context of VAIs’ care was largely negative, participants did describe examples where homecare workers provided compassionate care to the VAI, thereby improving the caregiving experience. Participants described examples of PSWs becoming friends with the care recipient who in turn provides the VAI with social support: *“Talking about friendships, my son often has a very “friendly relationship” with caregivers. It is so difficult to develop new friendships, so he usually tries to find caregivers with similar interests, sometime, he is lucky to have a caregiver who is a friend!”* (CG01). *“We actually have one nurse who's been with us for 19 years. He's become a very good friend. We do socialize a little with him and his wife, but we don't say anything to the agency that employs him because he really isn't supposed to do that.”* (MN01).

In addition to friendship, compassionate care was described by participants as entailing *“Good and consistent [care]”* (CG02). Having a consistent care team was described as being particularly important to participants given the amount of training they provide to healthcare professionals to feel comfortable with them providing care to the VAI. However, participants described that living in less urbanized areas frequently resulted in less consistent care providers.

#### Subtheme C: positive outlook on the caregiving journey

Participants described that their own positive outlook towards providing care allowed them to sustain the high level of care they continuously provide. Caregiving was sometimes described as giving meaning to their lives. One participant shared: *“I definitely feel like my life has more purpose/meaning and it really is a great feeling…motivating*” (CG07). Another participant described: *“I feel a lot of joy seeing that the fruits of my caregiving affect his life in a positive way, I feel happy from his big or small achievements. I guess life has more sense and meaning when one cares for a family member or another person”* (MN08). Participants also described that caregiving allowed them to get to know aspects of themselves better. One caregiver explicitly stated *“I have gotten to know myself better”* (CG02).

## Discussion

This study explored the experiences of caregivers and trained peer mentors during the pilot of an online peer support program that included live, weekly online text based chats. Findings highlight that caregivers to VAIs face unique challenges related to ventilator technology that may differ from other caregiving contexts, including a need for hypervigilance and challenges with boundary-setting and role overload. Sharing support with other caregivers was viewed as beneficial and several facilitators were identified that can make caregiving easier and joyful (i.e., social and tangible support from others, compassionate care, and a positive outlook). This information can be used to help advance future development of online peer support programs tailored to caregivers of VAIs.

Participants in this study highlighted the challenges of needing to provide constant care to VAIs. Coupled with the lack of training of healthcare providers to provide adequate care to VAIs, this substantially exacerbated their need to be hypervigilant. These finding highlights that the instrumental support received from homecare staff may not always meet caregivers’ actual needs (e.g., respite), even if they are meeting the needs of patients. As a result, caregiver participants reported declines in their own emotional, social, and physical health due to the difficulty of navigating the healthcare system to find support that meets their actual needs. These findings align with previous research that indicates that outside of specialist centres, community-based healthcare professionals tend to have a restricted understanding of ventilation, which limits how supported caregivers feel and ultimately compromises their well-being.^9^ The absence and inadequacy of community-based supports can generate additional responsibility and financial burden for caregivers as it becomes necessary from them to take on the majority of VAIs’ home-based care.^35^ Participants in our study described that they prioritized the needs of the VAI over their own unique needs, suggesting that improvements in caregiver well-being may be a secondary benefit of VAIs’ medical and health needs being met. In turn, investments in home care and ventilator-related training for providers are urgently needed to better support community-residing VAIs and their caregivers.

Our results add to the growing body of evidence pointing to the importance of online communities for supporting vulnerable caregivers who may not recognize their own needs for support as separate from the care needs of the VAI. Research has found that individuals with depression and less social support are more likely than other cohorts of caregivers to use peer support groups.^36^ Specific subtypes of social support (i.e., emotional, instrumental, informational, or appraisal support)^37^ should be incorporated into all peer support programs to potentially mitigate caregiver strain. Additionally, support is needed to help caregivers navigate the healthcare system to find health and community resources that appropriately meet their needs and contribute to their own well-being.^38^ Professional system navigator roles are increasingly being integrated into the healthcare system to aid patients and their caregivers with hospital to home transitions.^39^ Our group has previously identified health system-related challenges caregivers to VAIs experience, including a lack of access to referrals.^40^ The integration of system navigators in the context of ventilator-care may address gaps in caregiver navigational needs within the existing system. Our findings also suggest that despite the challenges of providing care to a VAI, there are facilitators that may help ease the caregiving experience. Future research is urged to consider the role that personal caregiver factors (e.g., competence in caregiving), support from family and friends, and presence of compassionate care may have in the mitigating the decline of caregiver health and well-being.

Our study extends the existing literature by suggesting that having a shared experience of caregiving for the same illness population, may supersede other types of similarity (e.g., caregiver-care recipient relationship; age; gender). In our study, support did not flow one way from trained mentors to peers, but instead, was reciprocal in nature. This reciprocal element of peer support can help sustain peer-support interventions.^41^ Future web-based peer support programs should optimize this reciprocity and ensure that there are ample opportunities for mentors and participants to share and receive support that meets their needs.^41^ In our study, the weekly chats were a good forum to generate shared conversations that allowed both trained mentors and caregiver participants to give and receive support. The online modality also appeared to equip caregivers with rapid and easily accessible peer support—especially to meet informational needs. Given the highly demanding nature of care that VAIs require, future online support programs should be flexible and allow attendance/use at the caregivers’ convenience.

A seminal theory on social support posits that individuals feel that others who have experienced the same situation are more likely to understand the way they handle situations and therefore are more likely to provide advice and support that resonates.^42^ We found this to be true in our study, with support from peers aligning with the goals outlined by the Peers for Progress framework—particularly information-sharing to support daily management of VAI care and system navigation advice to promote linkages with clinical care and community resources. We found this to be true regardless of whether participants had received formal training as mentors or not. Although existing research posits that training mentors provides them with knowledge that other participants lack,^41^ future research is merited to explore the influence extensive training has on the success of a peer support program. For instance, future implementation research, using rigorous study designs, may discern what benefit, if any, is derived from having trained peer mentors. This could be done by comparing the experiences of caregivers to VAIs in a peer support program with trained peer mentors, to one without.

Caregivers in our study were providing care to individuals who required ventilator assistance due to neuromuscular diseases (e.g., ALS, muscular dystrophy). There is no recovery from these diseases and although ventilation promotes prolonged survival, in many cases (particularly among patients with ALS), it marks an altered trajectory towards end of life. This has important implications for caregivers of VAIs who are likely to provide care for many years (9.7 and 14.5 years on average for caregivers and mentors in our study, respectively). The extended and highly demanding nature of care discussed by participants in our study underscores that future programs would benefit from facilitating ongoing support between peers that spans distances, care settings, and the prolonged trajectory of ventilator care. Further, our study delineates that care needs change over time, highlighting that future interventions would benefit from gaining insight into caregivers’ experiences at different points along the caregiving trajectory in order to ensure that these needs are being continuously met.^43,44^

## Strengths and limitations

By conducting a qualitative analysis of text chat data, we were able to gain insight into the naturally-occurring conversations that can take place between caregivers in online settings. An additional strength of our study was our use of a theoretical framework to guide the analysis and interpretation of data, thereby strengthening the potential for study findings to inform future design and implementation of peer support programming for this caregiving population. Some limitations of our study include the fact that all participants were from Ontario, Canada, spoke English, and had a great deal of experience with their caregiving role (mean duration of VAI care was 116 months). As a result, our findings may not reflect the experiences of less experienced, non-English speaking caregivers from other locations where the delivery of care for VAIs may differ. The majority of participants were also female, limiting our ability to capture the experiences of male caregivers and to make recommendations that can inform programming tailored to their needs. Finally, since this study focused on a web-based peer support program, it is limited in helping us understand what other modes of delivery may be useful for offering peer support to this caregiving population.

## Conclusion

The present study revealed that ventilator technology can result in unique challenges for VAI caregivers. However, sharing support with peers was viewed as beneficial and several facilitators were identified as making caregiving easier and joyful. Future support programs would benefit from creating opportunities for caregivers and trained peer mentors to reciprocally share support. Further, facilitating ongoing support across the caregiving trajectory has the potential to better-meet VAI caregivers’ changing needs as they provide prolonged care.
